# SYT7 acts as an oncogene and a potential therapeutic target and was regulated by ΔNp63α in HNSCC

**DOI:** 10.1186/s12935-021-02394-w

**Published:** 2021-12-20

**Authors:** You Fu, Guocai Tian, Zhiyuan Zhang, Xiao Yang

**Affiliations:** 1 Department of Oral and Maxillofacial-Head Neck Oncology, Shanghai Ninth People’s Hospital, Shanghai Jiao Tong University School of Medicine, College of Stomatology, National Center for Stomatology, National Clinical Research Center for Oral Diseases, Shanghai Jiao Tong University, 639 Zhizaoju Road, Shanghai, 200011 People’s Republic of China; 2 Department of Oral and Cranio-maxillofacial Surgery, Shanghai Ninth People’s Hospital, Shanghai Jiao Tong University School of Medicine, College of Stomatology, National Center for Stomatology, National Clinical Research Center for Oral Diseases, Shanghai Jiao Tong University, 639 Zhizaoju Road, Shanghai, 200011 People’s Republic of China; 3 Shanghai Key Laboratory of Stomatology, Research Unit of Oral and Maxillofacial Regenerative Medicine, Chinese Academy of Medical Sciences, Beijing, China

**Keywords:** ΔNp63 alpha, SYT7, Head and neck squamous cell carcinoma, TCGA, microarray

## Abstract

**Background:**

Head and neck squamous cell carcinoma (HNSCC) are one of the most common types of head and neck cancer, and it is urgent to find effective treatment for advanced patients. Exploring developing and progressing mechanisms of HNSCC could provide a theoretical basis to find new therapeutic targets.

**Methods:**

In our research, we performed a whole-gene expression profile microarray analysis to identify differential expression genes between squamous cell carcinoma cells and ΔNp63 alpha (ΔNp63α) knockdown cells. As a result, an important gene Synaptotagmin VII (SYT7) was screened out.

**Results:**

SYT7 knockdown affected the proliferation, apoptosis and cell cycle of squamous cell carcinoma cells. The rescue experiment in vitro with ΔNp63α and SYT7 double knockdown resulted in partial reversion of ΔNp63α-induced phenotypes. This was also confirmed by experiments in vivo.

**Conclusions:**

Taken together, we found that ΔNp63α could inhibit the occurrence and progression of HNSCC throughout downregulating the expression of SYT7. Therefore, SYT7/ΔNp63α axis could be a potential therapeutic target for clinical treatment of HNSCC.

**Supplementary Information:**

The online version contains supplementary material available at 10.1186/s12935-021-02394-w.

## Introduction

Head and Neck Cancer (HNC) is one of the ten most common malignant tumors worldwide. Advanced HNC patients may have various physiological dysfunctions and eating, speaking, breathing, audio-visual disorders, thus, seriously impacting health and life quality of patients. Head and neck squamous Cell Carcinoma (HNSCC) is the most common type of HNC, accounting for more than 90% of HNC. It is the sixth most common malignant tumor disease in the world [1, 2]. There are around 60,000 new cases every year, two-thirds of which are advanced patients at the III, IV stages [3, 4]. There are two thirds of HNSCC patients in developing countries. In China, HNSCC accounted for about 10% of the whole-body malignant tumors, and the trend of its incidence and fatality rate increased year by year. HNSCC generally originates from the head and neck mucosal epithelium, with strong metastasis and high recurrence rate. Therefore, HNSCC is very prone to postoperative recurrence and cervical lymph node metastasis. Given the poor prognosis of HNSCC and the lack of effective diagnosis and treatment [5], the 5-year survival rate of patients is only 50–60% [3, 6]. Therefore, it is particularly significant to explore the pathogenesis of HNSCC and to screen for specific diagnostic and prognostic biomarkers [7].

P53 transcription factor family, including p53 (TP53), p63 (TP63) and p73 (TP73), is a key player in tumor development and formation. p63 is expressed by two different promoters, resulting in two isoforms (TAp63 and DNp63) with or without amino (N) terminal transactivation domain that is necessary to induce apoptosis and tumor inhibition [8, 9]. Alternative splicing of the 3′ end of ΔNp63 mRNA produces α, β and γ subtype [10]. Among them, ΔNp63α is the most abundant subtype detected in the basal layer of mucosa, skin and other epithelial tissues, and is a transcription factor regulating the expression of genes involved in cell adhesion [11]. There are reports that ΔNp63α overexpression is associated with malignant diseases including squamous cell carcinoma, such as head and neck cancer and skin cancer [12]. However, the role of ΔNp63α in different tumor progression seems to be inconsistent. The study by Jiarong Chen et al. confirmed that ΔNp63α promotes the progression of lung squamous cell carcinoma by overexpressing LINC00173.v1 [13]. Ectopic expression of ΔNp63α in patients with intrauterine adhesions induces EMT and endometrial fibrosis [14]. Ying Zhou et al. pointed out that in cervical squamous cell carcinoma, ΔNp63α can exert anti-tumor ability by inhibiting cell migration, invasion and tumor growth [15]. Hitherto, the role and molecular mechanism of ΔNp63α in the progress of HNSCC are not clear enough, we need to further explore.

Synaptotagmins were identified as the most common Ca^2+^ sensors of cell exocytosis. Synaptotagmin VII (SYT7) was one of the members of the synaptotagmin family and was indispensable for facilitation at several central synapses [16]. SYT7 was presynaptic and could affinitively bind to Ca^2+^ and increase residual Ca^2+^ which regulated facilitation [17]. Previous studies showed that SYT7 played a significant role in the proliferation, migration, apoptosis, and cell cycle in multiple solid tumors, such as colorectal cancer, gastric cancer, non-small cell lung cancer, renal cell carcinoma, glioblastoma, and melanoma [16, 18–22]. However, the role of SYT7 in HNSCC stayed unclear. Revealing the functions and mechanisms of SYT7 in the progression and development of HNSCC could be of great value for the diagnosis, prognosis, and therapy for HNSCC patients.

In our study, we established ΔNp63α overexpressed cells in two HNSCC cell lines. Affymetrix expression profile assay was performed and a new important oncogene SYT7 was screened out. Rescue experiment showed that ΔNp63α and SYT7 double knockdown could partially reverse the SYT7-induced phenotype. In general, ΔNp63α could inhibit the occurrence and progression of HNSCC throughout downregulating the expression of SYT7. ΔNp63α and SYT7 could be potential targets and have promising therapeutic strategy for HNSCC patients.

## Materials and methods

### Cell culture

The human HNSCC cell lines, Cal-27 and HN6, were purchased from the Chinese Academy of Sciences Cell Bank/Stem Cell Bank. All cells were cultured in DMEM medium with high glucose (ThermoFisher, 11965126, USA) supplemented with 10% FBS (Gibco, 10100, USA) at 37 °C in a humidified atmosphere containing 5% CO_2_.

### Lentivirus‑mediated ΔNP63α overexpression, SYT7 knockdown and ΔNP63α, SYT7 double knockdown

The lentivirus-mediated short hairpin RNA (shRNA) vector system was designed, constructed, packed and purified by Shanghai GeneChem Co, Ltd. (Shanghai, China), and all procedures were performed according to the manufacturer’s protocol. Cal-27 and HN6 cells transfected with lentivirus, and ΔNP63α overexpression, shSYT7 and shSYT7 + shΔNP63α cells were established respectively and used for subsequent experiments. Cal-27 and HN6 cells transfected with blank lentivirus or lentivirus containing blank shRNA were used as a negative control, denoted as control group or shCtrl group. The multiplicity of infection was 10 (2 × 10^5^ cells transfected per well and 2 × 10^6^ TU lentivirus transfected per well in a 6-well plate), and the infection was proceeded with the addition of DMEM and 5 µg/mL Polybrene (Clontech Laboratories, Inc., Mountainview, CA, USA) to the cells. The fluorescent microscopy (Olympus IX71; Olympus Corporation, Tokyo, Japan) demonstrated that the transfection efficiency of Cal-27 and HN6 cells transfected with gene-overexpressed, gene-shRNA and shCtrl lentivirus was > 80%. The overexpression or knockdown efficiency of the target gene was detected by RT-qPCR and western blotting 3 days after transfection.

### RNA sequencing and data analysis

Total RNA from HN6 cells transfected with shΔNP63α and shCtrl lentivirus was extracted using Trizol Reagent (Life technologies, Carlsbad, CA, US) according to the standard operating procedure provided by the manufacturer, and was further purified by QIAGEN RNeasy Kit. The total RNA was amplified, labeled and purified by using GeneChip® 3′ IVT PLUS Reagent Kit (Affymetrix, Santa Clara, CA, US) followed standard operating procedure to obtain the biotin labeled cRNA. According to the hybridization standard process provided by Affymetrix expression profile chip, array hybridization was conducted by GeneChip® Hybridization, Wash and Stand Kit (Affymetrix). The chip results were scanned with GeneChip® Scanner 3000 (Affymetrix), and the original data was read by Command Console Software 4.0 (Affymetrix). The qualified data were normalized by Affy package in R software. Limma package in R software was used for differential gene screening, and the selection conditions were as follows: Fold Change ≤ 0.5 or Fold Change ≥ 2, and *P* < 0.05. Then, the clusterProfiler package in R/Bioconductor was used to perform Gene Ontology (GO) analysis and Kyoto Encyclopedia of Genes and Genomes (KEGG) enrichment analysis based on the differentially expressed genes for further function and pathway enrichment analysis. Ingenuity Pathway Analysis (IPA) was used to analyze the interaction network between target gene and significantly enriched pathways and pathway-related differentially expressed molecules.

### Real-time polymerase chain reaction (PCR) analysis

Total RNA was extracted using TRIzol reagent (Invitrogen, USA), and cDNA was synthesized using SuperScript IV (Invitrogen, USA) following the manufacturer’s instructions. The expression of target gene mRNA was measured by qRT-PCR (StepOnePlus™ Real-Time PCR System, Thermo Fisher, 4376600) using SYBR Green technology. Thermocycling conditions were as follows: 95 °C for 60 s, then 45 cycles of 95 °C for 10 s, 60 °C for 30 s). The forward and reverse primer sequences were provided in Additional file 1: Table S1. The average cycle threshold (CT) value of the target gene in each group was calculated, and the CT value of the internal reference gene was subtracted to determine the ΔCT value. The average ΔCT value of the control group was calculated, and the ΔCT value of each group sample was subtracted to determine the −ΔΔCT value. The expression of gene mRNA was calculated by using the 2^−ΔΔCT^ method. GAPDH was the internal reference gene. The values for the control group were set as 1, and the values for the other groups were calculated as the fold changes relative to the control values.

### Western blot analysis

Western blotting was used to detect the protein level in Cal-27 and HN6 cells. Proteins were extracted from cells using a lysis buffer (2% 2-mercaptoethanol, 4% SDS and 20% glycerol, 100 mM Tris-HCl), and the protein concentration was measured using a bicinchoninic acid (BCA) Protein Assay kit (Beyotime Institute of Biotechnology, Haimen, China). A total of 20 µg protein was separated by SDS-PAGE (10% gels), and transferred onto polyvinylidene fluoride membranes (EMD Millipore, Billerica, MA, USA). The membranes were blocked in Tris-buffered saline with Tween (TBST) containing 5% non‑fat milk overnight at 4 °C. The membranes were then incubated with primary antibodies overnight at 4 °C. The membranes were then washed three times in TBST. The membranes were incubated with a horseradish peroxidase-conjugated goat anti-mouse immuno-globulin secondary antibody for 2 h at room temperature, then washed three times in TBST. Bands were visualized using enhanced chemiluminescence (ECL; Pierce; Thermo Fisher Scientific, Inc.), according to the manufacturer’s protocol. The antibodies were provided in Additional file 2: Table S2.

### Celigo analysis

Cells were seeded at a density of 2.5 × 10^3^ cells/well in a 96-well plate at 72 h post-transfection (at 37 °C in an atmosphere of 5% CO_2_). After plating, Celigo® Image Cytometer (Nexcelom, Lawrence, MA, USA) was used to evaluate the number of cells by scanning green fluorescence daily for 5 days at room temperature.

### MTT assay

Cells transfected with lentivirus were seeded in the 96-well culture plates at 2000 cells per well. The medium was replaced once a day and a 96-well plate was selected for MTT determination for 5 consecutive days. 20 µL MTT solution (Genview) was added into each well and incubated for 4 h. 100 µL DMSO solution was added into each well and shaken on a shaker for 2–5 min. Absorbance at 490 nm was recorded using the SpectraMax M5 microplate reader (Molecular Devices, Silicon Valley, CA, USA).

### Wound-healing assay

5 × 10^4^ Cal-27 and HN6 cells transfected with lentivirus were inoculated into 96-well plates and cultured at 37 °C in an incubator with 5% CO_2_, with 3 replicate wells per group. On the next day, 96 Wounding Replicator (VP408FH, VP scientific) was aimed at the center of the lower end of the 96-well plate, gently pushed up to form a scratch, and the plates were washed 2–3 times with serum-free medium. 0.5% FBS-containing medium was added to the 96-well plate, and the plate was scanned with Cellomics (ArrayScan VT1, Thermo) to obtain 0 h pictures. After 8 h, the plate was scanned with Cellomics again to get the picture. The cell area was calculated and the cell migration ability was evaluated.

### Flow cytometry analysis for apoptosis

Fluorescence-activated cell sorting (FACS) was used to analyze the lentiviral efficiency at cell apoptosis. Cells transfected with lentivirus were plated in 6-cm dishes 5 days after transfection and grown to 85% confluence. Following washing with binding buffer [from the Annexin V-allophycocyanin (APC) Detection kit (cat. no. 88-8007; eBioscience; Thermo Fisher Scientific, Inc.)] once, cells were stained with 200 µL binding buffer containing 10 µL APC Detection kit for 10–15 min at room temperature in the dark. A flow cytometer (Merck KGaA) and InCyte 3.1 (Merck KGaA) were then used to analyze the cells.

### Flow cytometry analysis for cell cycle

Fluorescence-activated cell sorting (FACS) was performed to analyze the cell cycle distribution of CAL-27 and HN6 cells after transfection with lentivirus. Cells transfected with lentivirus were seeded in 6-cm dishes and grown to a coverage rate of approximately 80%. After washing once with PBS pre-cooled at 4 °C, the cells were fixed with 70% ethanol at 4 °C for 1 h, and then washed once with PBS. The cell staining solution was prepared in the ratio of 25:10:1000 (40 × PI (2 mg/mL, P4170, Sigma): 100 × RNase (10 mg/mL, 2158-1, TakaRa): 1 × PBS). The cells were resuspended with a certain volume of cell staining solution, and then analyzed using a flow cytometer (Merck KGaA).

### Immunohistochemical staining

Tumor samples of patients with HNSCC were obtained between 2006 and 2018 from Shanghai Ninth People’s Hospital. All patients signed the informed consent form. All the tumor samples were fixed with 4% paraformaldehyde (Sangon Biotech) and embedded in paraffin. 4‑µm‑thick slides were performed and used for subsequent staining. Slides were incubated in 3% H_2_O_2_ for 5 min to block endogenous peroxidase was with, and then blocked with 5% serum for 15 min. Slides were incubated with primary antibodies (Abs) at 37 °C for 2 h, and with secondary antibody at 37 °C for 45 min. The samples were counterstained with hematoxylin (Absin Bioscience, Inc, abs9139) for 10–15 s. Finally, after dehydration, the slides were mounted with neutral gum. All IHC images were examined with a microscope (ZEISS Axioscope 5).

### Animal experiments

Female BALB/c nude mice aged 4 weeks were purchased from the Charles River Laboratories. All mice were maintained under specific pathogen-free conditions in the animal facilities of the Ninth People’s Hospital, and were divided into two groups at random. Cal-27 cells transfected with shSYT7 or shCtrl lentivirus were implanted by subcutaneous injection of 2 × 10^6^ cells underneath the skin of right back. Tumor sizes were calculated as length × width × width × 0.5. Before the mice were sacrificed, the mice were anesthetized with pentobarbital (Sigma‑Aldrich, USA) at a dose of 80 mg/kg (injected intraperitoneally), and then the fluorescence intensity in the nude mice was observed and photographed using a small animal in vivo imaging system (LB983, Berthold technologies). Mice with volumes greater than 2000 mm^2^ were euthanized with 60% compressed CO_2_ gas at a flow rate of 20% chamber vol/min. All animal experiments were approved by and performed in accordance with the guidelines of the Shanghai Jiao Tong University School of Medicine.

### Statistical analysis

The data were shown as the mean ± SD. One-way or two-way analysis of variance (ANOVA) followed by Tukey’s post hoc analysis was performed to identify any significant differences. A computer-based statistical package (SPSS, version 22.0) was utilized for the analysis and Graphpad prism version 8.0 was used for the production of statistical graphs. *P *< 0.05 was regarded to indicate a statistically significant difference.

## Results

### Overexpression of ΔNp63α affects proliferation, apoptosis and cell cycle of HNSCC cell lines

**Fig. 1:**
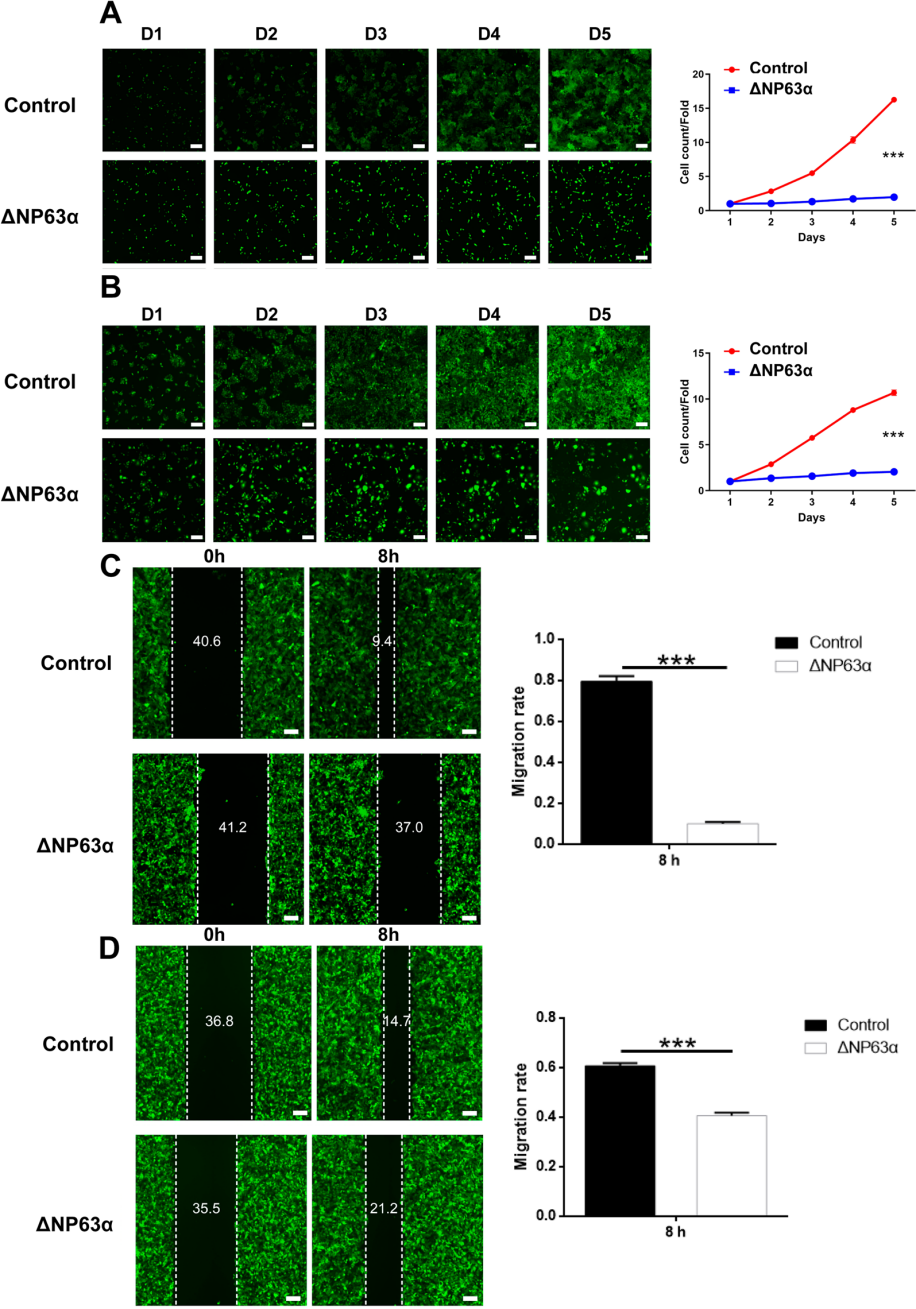
Overexpression of ΔNP63α inhibited growth and migration of HN6 and CAL-27 cells.** A** Detection of cell proliferation of HN6 cells by Celigo cell count. **B** Detection of cell proliferation of CAL-27 cells by Celigo cell count. **C** Wound-healing assay of HN6 cells. **D** Wound-healing assay of CAL-27 cells (n = 3). Bars show the mean ± SD. ***Represents *p *< 0.001. Scale bar = 100 μm

**Fig. 2:**
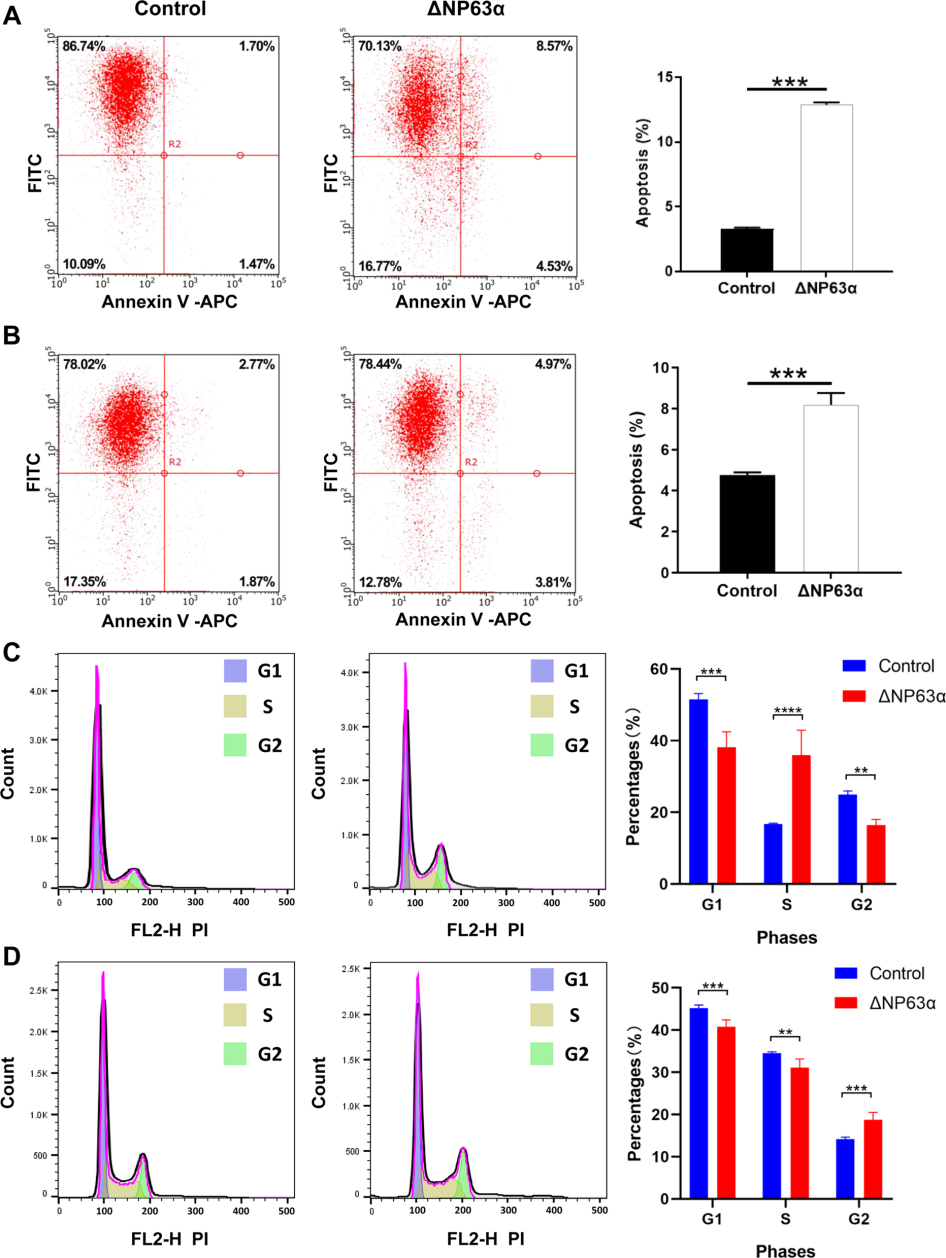
Overexpression of ΔNP63α induced cell apoptosis and reduced the proportion of tumor cells in G1 phase in HN6 and CAL-27 cells.** A** Apoptosis assay of HN6 cells. **B** Apoptosis assay of CAL-27 cells. **C** Cell cycle detection of HN6 cells. **D** Cell cycle detection of CAL-27 cells (n = 3). Bars show the mean ± SD. **Represents *p *< 0.01, ***represents *p *< 0.001

We selected human HNSCC cell lines HN6 and CAL-27 and established ΔNp63α-overexpressed cells (Additional file 3: Fig. S1). ΔNp63α mRNA and protein levels were memorably upregulated in HN6 and CAL-27 cells transfected with ΔNp63α-overexpressed lentivirus (Additional file 3: Fig. S1I–L). Celigo assays demonstrated that overexpression of ΔNp63α could significantly suppress the proliferation of HNSCC cells in vitro (Fig. 1A, B). Analogously, MTT assays also verified this result (Additional file 4: Fig. S2A, B). Wound-healing assays showed that overexpression of ΔNp63α also inhibited the migration of HNSCC cells (Fig. 1C, D). Then, flow cytometry was performed to the influence of overexpression of ΔNp63α on cell cycle and apoptosis. Results showed that overexpression of ΔNp63α could lead to increasing apoptosis of HNSCC cells (Fig. 2A, B) and significantly reduce the proportion of tumor cells in G1 phase (Fig. 2C, D).

### SYT7 was screened as a target gene of ΔNp63α and an oncogene in HNSCC

**Fig. 3:**
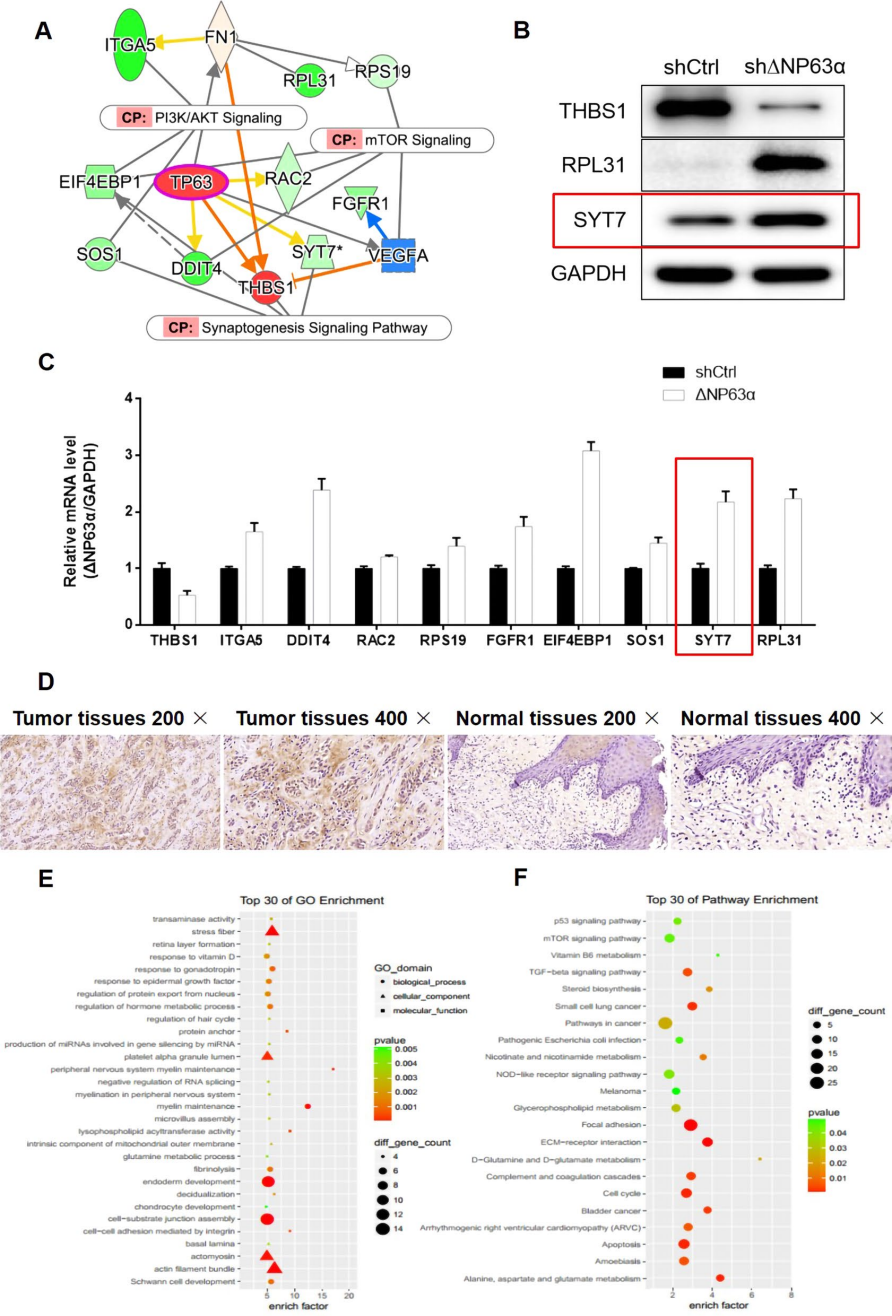
Bioinformatic analysis of ΔNP63α-knockdown cells.** A** IPA analysis of ΔNP63α-knockdown cells. **B** Protein expression of between shCtrl cells and ΔNP63α-knockdown cells. **C** mRNA expression of between shCtrl cells and ΔNP63α-knockdown cells. **D** Microarray detection of SYT7 expression in HNSCC samples and paired normal tissues. **E** GO enrichment of differential genes. **F** KEGG pathway enrichment of differential genes

To further clarify the downstream target genes of ΔNp63α, we performed Transcriptional sequencing on ΔNp63α-knockdown cells and shCtrl cells. We identified several related genes through IPA analysis (Fig. 3A). Genes differentially expressed in ΔNp63α-knockdown cells were detected at the protein and mRNA levels (Fig. 3B, C). Among them, SYT7 not only had a direct interaction with ΔNp63α, but also was significantly enhanced at the mRNA and protein levels in ΔNp63α-knockdown cells, thereby it was selected as the downstream target gene of ΔNp63α to explore the role in HNSCC cell proliferation and migration.

Oral squamous cell carcinoma (OSCC) accounts for more than 90% of all histological subtypes of oral cancer, and is highly aggressive [23]. In this study, the results of immunohistochemical staining revealed that the levels of SYT7 in OSCC tissues was markedly higher that than in para-carcinoma tissues (Fig. 3D; Table 1). Further statistical analysis suggested that the expression of SYT7 gene was significantly different in the pathological grading of OSCC (Table 2), and there was a positive correlation (Table 3). Moreover, GO and KEGG enrichment was also conducted. Differentially expressed genes were mainly enriched in GO functions such as stress fiber, actomyosin, and actin filament bundle (Fig. 3E), and KEGG signaling pathways such as p53 signaling pathway, mTOR signaling pathway, Pathways in cancer, Cell cycle, and Apoptosis (Fig. 3F).

**Table 1:** Expression patterns in oral squamous cell carcinoma tissues and para-carcinoma tissues revealed in immunohistochemistry analysis

SYT7 expression	Tumor tissue		Para-carcinoma tissue		*p* value
	Cases	Percentage (%)	Cases	Percentage (%)	
Low	27	55.0	8	100.0	< 0.001***
High	33	45.0	0	0.0	

**Table 2:** Relationship between SYT7 expression and tumor characteristics in patients with oral squamous cell carcinoma

Features	No. of patients	SYT7 expression		*p* value
		Low	High	
All patients	60	27	33	
Age (years)				0.737
≤ 64	31	14	17	
> 64	27	11	16	
Gender				0.486
Male	28	11	17	
Female	31	15	16	
Grade				< 0.001***
1	22	18	4	
2	28	6	22	
3	7	0	7	
Tumor size (cm)				0.757
< 2.5	22	8	14	
≥ 2.5	27	11	16	
Lymph node positive				0.129
= 0	25	13	12	
> 0	9	2	7	
Lymphatic metastasis (N)				0.129
N0	25	13	12	
N1	9	2	7	

**Table 3:** Relationship between SYT7 expression and tumor characteristics in patients oral squamous cell carcinoma

		SYT7
Grade	Spearman correlation	0.643
	Significance (two-tailed)	< 0.001***
	N	57

### Knockdown of SYT7 affected the proliferation and migration of HNSCC cells and the growth of tumor in vivo

**Fig. 4:**
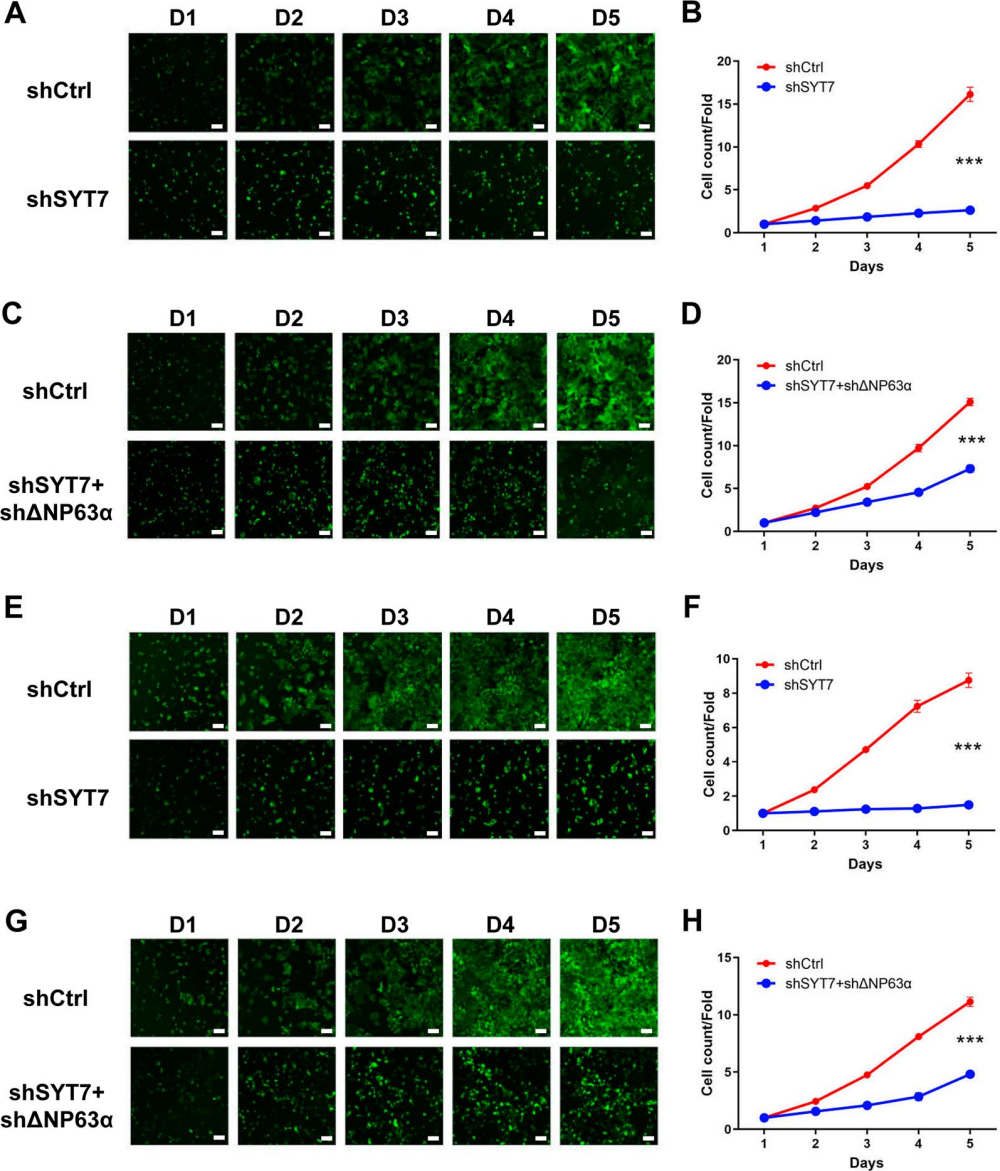
Knockdown of SYT7 suppressed the proliferation of HN6 and CAL-27 cells and ΔNP63α-SYT7 double knockdown could partially rescue this phenotype. **A**, **B** Detection of cell proliferation of SYT7-knockdown HN6 cells by Celigo cell count. **C**, **D** Detection of cell proliferation of ΔNP63α SYT7 double knockdown HN6 cells by Celigo cell count. **E**, **F** Detection of cell proliferation of SYT7-knockdown CAL-27 cells by Celigo cell count. **G**, **H** Detection of cell proliferation of ΔNP63α SYT7 double knockdown CAL-27 cells by Celigo cell count (n = 3). Bars show the mean ± SD. ***Represents p < 0.001. Scale bar = 100 μm

**Fig. 5:**
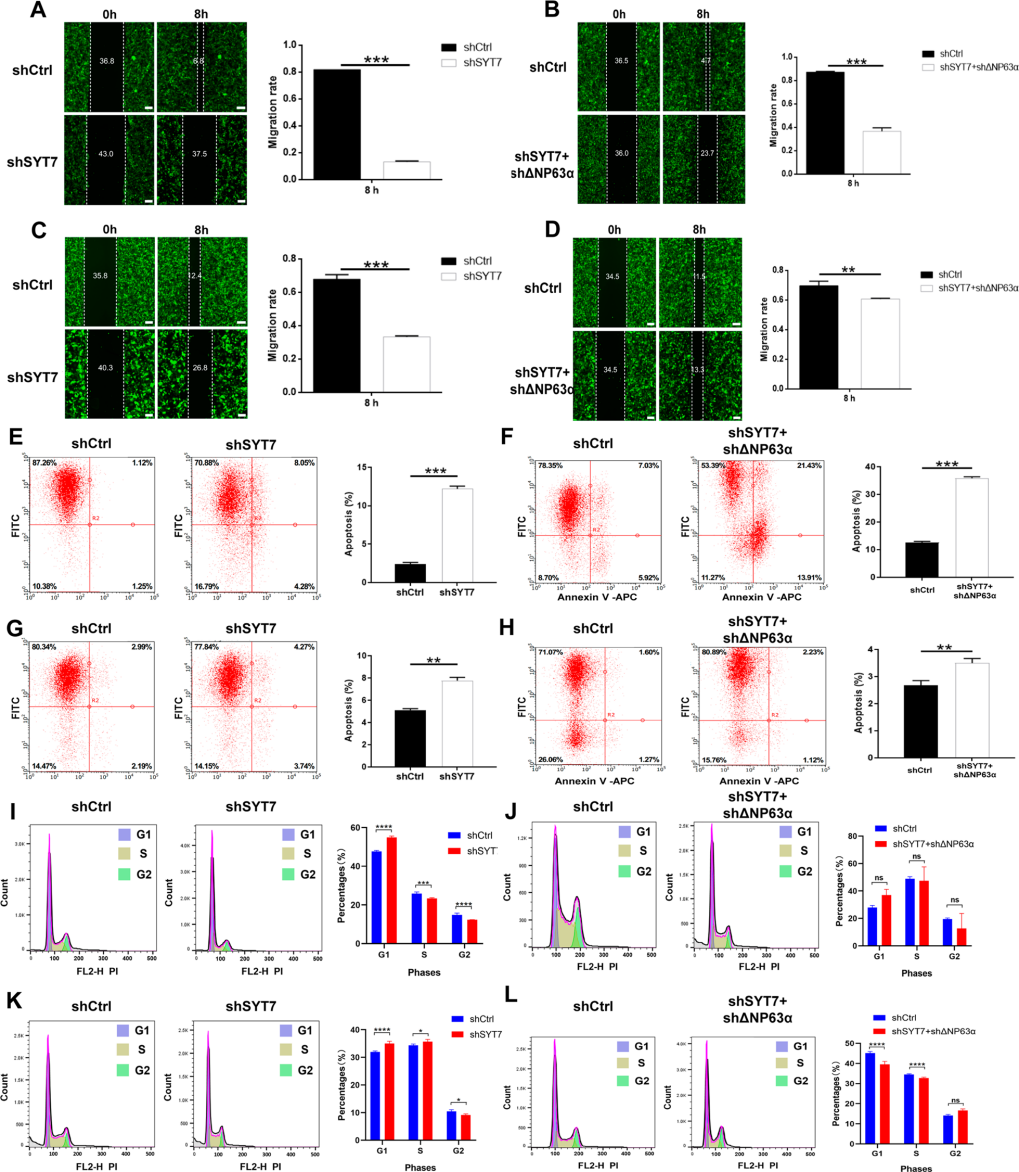
Knockdown of SYT7 induced cell apoptosis and influenced cell cycle of HN6 and CAL-27 cells and ΔNP63α-SYT7 double knockdown could partially rescue this phenotype.** A** Wound-healing analysis of SYT7-knockdown HN6 cells. **B** Wound-healing analysis of ΔNP63α SYT7 double knockdown HN6 cells. **C** Wound-healing analysis of SYT7-knockdown CAL-27 cells. **D** Wound-healing analysis of ΔNP63α SYT7 double knockdown CAL-27 cells. **E** Apoptosis assay of SYT7-knockdown and shCtrl HN6 cells. **F** Apoptosis assay of ΔNP63α SYT7 double knockdown and shCtrl HN6 cells. **G** Apoptosis assay of SYT7-knockdown and shCtrl CAL-27 cells. **H** Apoptosis assay of ΔNP63α SYT7 double knockdown and shCtrl CAL-27 cells. **I** Cell cycle detection of SYT7-knockdown and shCtrl HN6 cells. **J** Cell cycle detection of ΔNP63α SYT7 double knockdown and shCtrl HN6 cells. **K** Cell cycle detection of SYT7-knockdown and shCtrl CAL-27 cells. **L** Cell cycle detection of ΔNP63α SYT7 double knockdown and shCtrl CAL-27 cells (n = 3). Bars show the mean ± SD. *Represents p < 0.05, **represents p < 0.01, ***represents p < 0.001, ****represents p < 0.0001. Scale bar = 100 μm

**Fig. 6:**
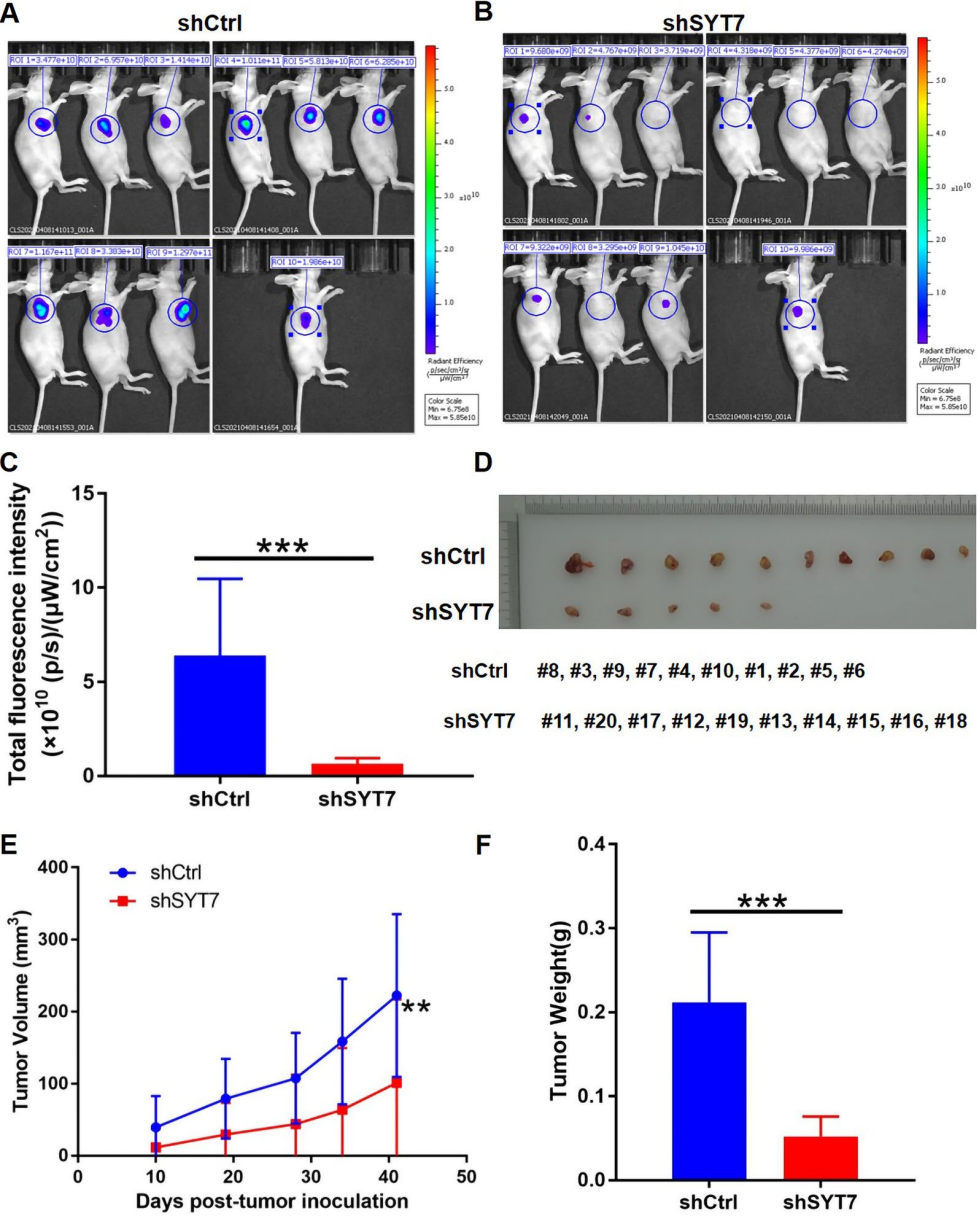
Establishment of SYT7-knockdown xenograft tumor model in nude mice.** A**–**C** In vivo imaging analysis of nude mice after SYT7 knockdown. **D** Tumor samples from nude mice. **E** Tumor growth curve in nude mice with SYT7 knockdown. **F** Weight of tumors collected from nude mice (n = 10). Bars show the mean ± SD. **Represents p < 0.01, ***represents p < 0.001

To identify the role of SYT7 in HNSCC, we established SYT7-knockdwon, in which SYT7 mRNA and protein levels were obviously diminished (Additional file 5: Fig. S3A–F). Besides, SYT7-ΔNp63α double knockdown cells was also constructed to study the effects of ΔNp63α and SYT7 interaction on HN6 and CAL-27 cells (Additional file 5: Fig. S3G–N). Celigo assays have been performed and results showed that knockdown of SYT7 markedly suppressed the proliferation of HNSCC cells in vitro (Fig. 4A, B, E, F), while SYT7-ΔNp63α double knockdown could partially rescue this phenotype (Fig. 4C, D, G, H). Wound-healing assays showed that knockdown of SYT7 also inhibited the migration of HNSCC cells (Fig. 5A, C), and SYT7-ΔNp63α double knockdown could partially alleviate the inhibition of HNSCC cell migration by SYT7 downregulation (Fig. 5B, D). Then, flow cytometry was performed to the influence of SYT7-knockdwon and SYT7-ΔNp63α double knockdown on cell cycle and apoptosis. It was found that knockdown of SYT7 could clearly inducing apoptosis of HN6 and CAL-27 cells, while SYT7-ΔNp63α double knockdown could partly restrict the induction of apoptosis of CAL-27 cell by shSYT7 lentivirus (Fig. 5E–H). Results showed that knockdown of SYT7 could also influence cell cycle of tumor cells (Fig. 5I–L). Furthermore, we also established a SYT7 knockdown HNSCC xenograft tumor model. In vivo imaging results indicated that the total fluorescence intensity in nude mice after SYT7 knockdown was lower than that of the control group (Fig. 6A–C). In addition, the volume and weight of tumors in the SYT7 knockdown group were strikingly reduced than those in the control group, suggesting that SYT7 knockdown significantly suppressed tumor growth in vivo (Fig. 6D–F).

## Discussion

HNSCC is characterized as anatomical site complexity, intraneoplastic heterogeneity, genetic diversity among individuals [24, 25]. Drug resistance, recurrence and metastasis is the most common death cause of HNSCC [26, 27]. Due to the lack of more effective diagnostic and treatments, it is imperative to screen specific diagnostic and prognostic biomarkers. Our data indicated that ΔNp63α overexpression retarded the proliferation and migration of HNSCC cell lines (HN6 and CAL-27), and induced cell apoptosis. As a downstream target gene, SYT7 was highly expressed in HNSCC tissues and augmented by ΔNp63α knockdown. SYT7 silencing slowed down the proliferation and migration of HNSCC cells, and increased the rate of apoptosis.

Previous studies have shown that ΔNp63α was overexpressed and associated with poor prognosis and survival, and partly triggered its tumorigenic effect by promoting proliferation and cell survival [28]. ΔNp63α activated the Wnt/β-catenin signaling pathway through transcriptional regulation of RSK4, which played a key role in the progression and radioresistance of esophageal squamous cell carcinoma [29]. In addition, ΔNp63α inhibited the PKCγ/Rac1 signaling pathway by positively regulating miR-320a, thereby inhibiting cancer invasion [30]. In our study, overexpression of ΔNp63α suppressed the proliferation and migration of HN6 and CAL-27 cells, induced apoptosis and affected the distribution of cell cycle, suggesting that ΔNp63α played a tumor suppressor effect in HNSCC. However, in the study of Jacky Chuang et al., ΔNp63α was a key pro-survival protein, which was overexpressed in 80% of HNSCC and enhanced the resistance of HNSCC to chemotherapy-induced cell death [31]. Therefore, the role of ΔNp63α in the progression of HNSCC needs further verification.

As the main isotype of the transcription factor TP63 expressed in squamous cell carcinoma, ΔNp63α played a tumor-promoting effect in squamous cell carcinoma by regulating the expression of different target genes [32]. In this study, it was confirmed that there was an interaction relationship between SYT7 and ΔNp63α through Affymetrix expression profile assay and IPA and other bioinformatic analysis, and ΔNp63α knockdown boosted the levels of SYT7 mRNA and protein. SYT7 was a Ca^2+^ sensor which had a variety of functions and expressed throughout the whole body. SYT7 had great affinity to Ca^2+^ and played a significant role in the process of modest calcium increased [33]. In non-small cell lung cancer, the expression of SYT7 was distinctly higher than that of para-carcinoma tissues, and the high expression of SYT7 protein was related to the low survival rate of patients [20]. Moreover, compared with normal tissues, the levels of SYT7 in colorectal cancer tissues were up-regulated and were positively correlated with pathological staging. Knockdown of SYT7 gene inhibited cell proliferation and clonal formation, and promoted G2/M phase arrest and apoptosis [21]. In the study, the expression level of SYT7 in OSCC tumor tissues was higher than that in para-carcinoma tissues. The expression of SYT7 gene was significantly different in the pathological grading of OSCC, and there was a positive correlation.

Mitsuro Kanda et al. determined that SYT7 was highly expressed in gastric cancer tissues with liver metastases. SYT7 gene knockdown inhibited GC progression and metastasis, which was manifested by increased apoptosis and weakened cell migration, invasion and adhesion [22]. In our study, we established SYT7 knockdown cells in two HNSCC cell lines, HN6 and CAL-27. Results showed that knockdown of SYT7 remarkably inhibited proliferation and migration, and induced apoptosis and cell cycle. Nevertheless, this phenotype could be rescued by ΔNp63α and SYT7 double knockdown. These results demonstrated that SYT7 was an oncogene in HNSCC and regulated by ΔNp63α. However, there are still some shortcoming in this study. We should detect the expression levels of ΔNp63α in HNSCC tissues and para-carcinoma tissues. Besides, we did not further evaluate the correlation between the expression of SYT7 in HNSCC and tumor pathological characteristics by statistical analysis.

## Conclusions

In summary, our study demonstrated that ΔNp63α played a role in HNSCC as a tumor suppressor gene, and SYT7 played a significant role in cell proliferation, migration, apoptosis and cell cycle of HNSCC tumor cells under the regulation of ΔNp63α. We first reported SYT7 as an oncogene and a potential diagnostic and prognostic biomarker in HNSCC, thus, SYT7 was considered to be a promising diagnostic and therapeutic target for HNSCC patients.

## Supplementary Information

**Additional file 1: Table S1.** Primers used in qPCR.

**Additional file 2: Table S2.** Antibodies used in westernblotting and IHC.

**Additional file 3: Figure S1.** Establishment of ΔNP63α-overexpressed cells.** A**–**D** The fluorescence observation of ΔNP63α-overexpressed HN6 cells. **E**–**H** The fluorescence observation of ΔNP63α-overexpressed CAL-27 cells. **I** The expression levels of ΔNP63α mRNA in ΔNP63α-overexpressed HN6 cells. **J** The expression levels of ΔNP63α mRNA in ΔNP63α-overexpressed CAL-27 cells. **K** The expression levels of ΔNP63α protein in ΔNP63α-overexpressed HN6 cells. **L** The expression levels of ΔNP63α protein in ΔNP63α-overexpressed CAL-27 cells (n = 3). Bars show the mean ± SD. *Represents p < 0.05, **represents p < 0.01. Scale bar = 100 μm.

**Additional file 4: Figure S2.** Cell proliferation analysis of ΔNP63α-overexpressed cells.** A** MTT assay of HN6 cells. **B** MTT assay of CAL-27 cells (n = 3). Bars show the mean ± SD. ***Represents p < 0.001.

**Additional file 5: Figure S3.** Establishment of SYT7-knockdown cells and ΔNP63α SYT7 double knockdown cells.** A** The fluorescence observation of SYT7-knockdown HN6 cells. **B** The fluorescence observation of SYT7-knockdown CAL-27 cells. **C** The expression levels of SYT7 mRNA in SYT7-knockdown HN6 cells. **D** The expression levels of SYT7 protein in SYT7-knockdown HN6 cells. **E** The expression levels of SYT7 mRNA in SYT7-knockdown CAL-27 cells. **F** The expression levels of SYT7 protein in SYT7-knockdown CAL-27 cells. **G** The fluorescence observation of ΔNP63α SYT7 double knockdown HN6 cells. **H** The fluorescence observation of ΔNP63α SYT7 double knockdown CAL-27 cells. **I**–**J** The expression levels of ΔNP63α and SYT7 mRNA in ΔNP63α SYT7 double knockdown HN6 cells. **K**–**L** The expression levels of ΔNP63α and SYT7 mRNA in ΔNP63α SYT7 double knockdown CAL-27 cells. **M** The expression levels of ΔNP63α and SYT7 protein in ΔNP63α SYT7 double knockdown HN6 cells. **N** The expression levels of ΔNP63α and SYT7 protein in ΔNP63α SYT7 double knockdown CAL-27 cells (n = 3). Bars show the mean ± SD. *Represents p < 0.05, **represents p < 0.01, ***represents p < 0.001. Scale bar = 100 μm.

## Data Availability

All data generated or analyzed during this study are included in this published article or are available from the corresponding author on reasonable request.
