# Supplementary material for: SYT7 acts as an oncogene and a potential therapeutic target and was regulated by ΔNp63α in HNSCC
Source: Cancer Cell Int. 2021 Dec 20;21:696. doi: 10.1186/s12935-021-02394-w (PMC8691088; doi:10.1186/s12935-021-02394-w)
Supplement: Supplementary file 1 — Additional file 1: Table S1. Primers used in qPCR. [file 12935_2021_2394_MOESM1_ESM.docx]

**Table S1.** Primers used in qPCR

| Gene | Forward primer sequence (5'-3') | Reverse primer sequence (5'-3') |
| --- | --- | --- |
| ∆NP63α | GCCGTGAGACTTATGAAATGC | TCTGAAGTAAGTGCTGGTGCTG |
| THBS1 | TGTTCTCTACTGGCTTTATGTCA | GGCCTGAGCAACTCAGTCTT |
| ITGA5 | GACCCAGCAGGGAGTAGTGTTT | GAAGGGCAGAGCCAAAGAA |
| DDIT4 | TTAGCAGTTCTCGCTGACCG | CCAAAGGCTAGGCATGGTGA |
| RAC2 | TCGTCAGCCCAGCCTCTTAT | TCAGTTTCTCGATGGTGTCCTT |
| RPS19 | CGAATGGGTGGATACCGTCA | TCACCAGCTCGCGTGTAGAA |
| FGFR1 | TGCCCGCCAACAAAACA | AATCTTGCTCCCATTCACCTC |
| EIF4EBP1 | ACTCACCTGTGACCAAAACACC | CCGCTTATCTTCTGGGCTATT |
| SOS1 | GCCAGCCTCATTGTCCCTAA | ACTGAAGGGGGTCCAATGTG |
| SYT7 | ATCTACCTGCTGCCCGACAA | CCTCTGCACCACCTTCTCA |
| RPL31 | GCTCAACAAAGCTGTCTGGG | TTATGGGCTCTTGGCGACT |
| GAPDH | TGACTTCAACAGCGACACCCA | CACCCTGTTGCTGTAGCCAAA |
