# Supplementary material for: SYT7 acts as an oncogene and a potential therapeutic target and was regulated by ΔNp63α in HNSCC
Source: Cancer Cell Int. 2021 Dec 20;21:696. doi: 10.1186/s12935-021-02394-w (PMC8691088; doi:10.1186/s12935-021-02394-w)
Supplement: Supplementary file 2 — Additional file 2: Table S2. Antibodies used in westernblotting and IHC. [file 12935_2021_2394_MOESM2_ESM.docx]

**Table S2.** Antibodies used in western blotting and IHC

| Primary antibodies | Dilution in WB | Source species | Company | Catalog No. |
| --- | --- | --- | --- | --- |
| △NP63α | 1:1000 | Rabbit | CST | 67825 |
| THBS1 | 1:1000 | Rabbit | CST | 37879 |
| RPL31 | 1:1000 | Rabbit | abcam | Ab103991 |
| SYT7 | 1:1000 | Rabbit | Tianjin Saierbio | SRP09489 |
| GAPDH | 1:3000 | Rabbit | Bioworld | AP0063 |
| Primary antibodies | Dilution in IHC | Source species | Company | Catalog No. |
| SYT7 | 1:50 | Rabbit | Tianjin Saierbio | SRP19489 |
| Ki67 | 1:300 | Rabbit | abcam | Ab16667 |
| Secondary antibody | Dilution |  | Company | Catalog No. |
| HRP Goat Anti-Rabbit IgG (WB) | 1:3000 |  | Beyotime | A0208 |
| HRP Goat Anti-Rabbit IgG (IHC) | 1:400 |  | Abcam | Ab97080 |
