# Supplementary figures and images for: SYT7 acts as an oncogene and a potential therapeutic target and was regulated by ΔNp63α in HNSCC
Source: Cancer Cell Int. 2021 Dec 20;21:696. doi: 10.1186/s12935-021-02394-w (PMC8691088; doi:10.1186/s12935-021-02394-w)

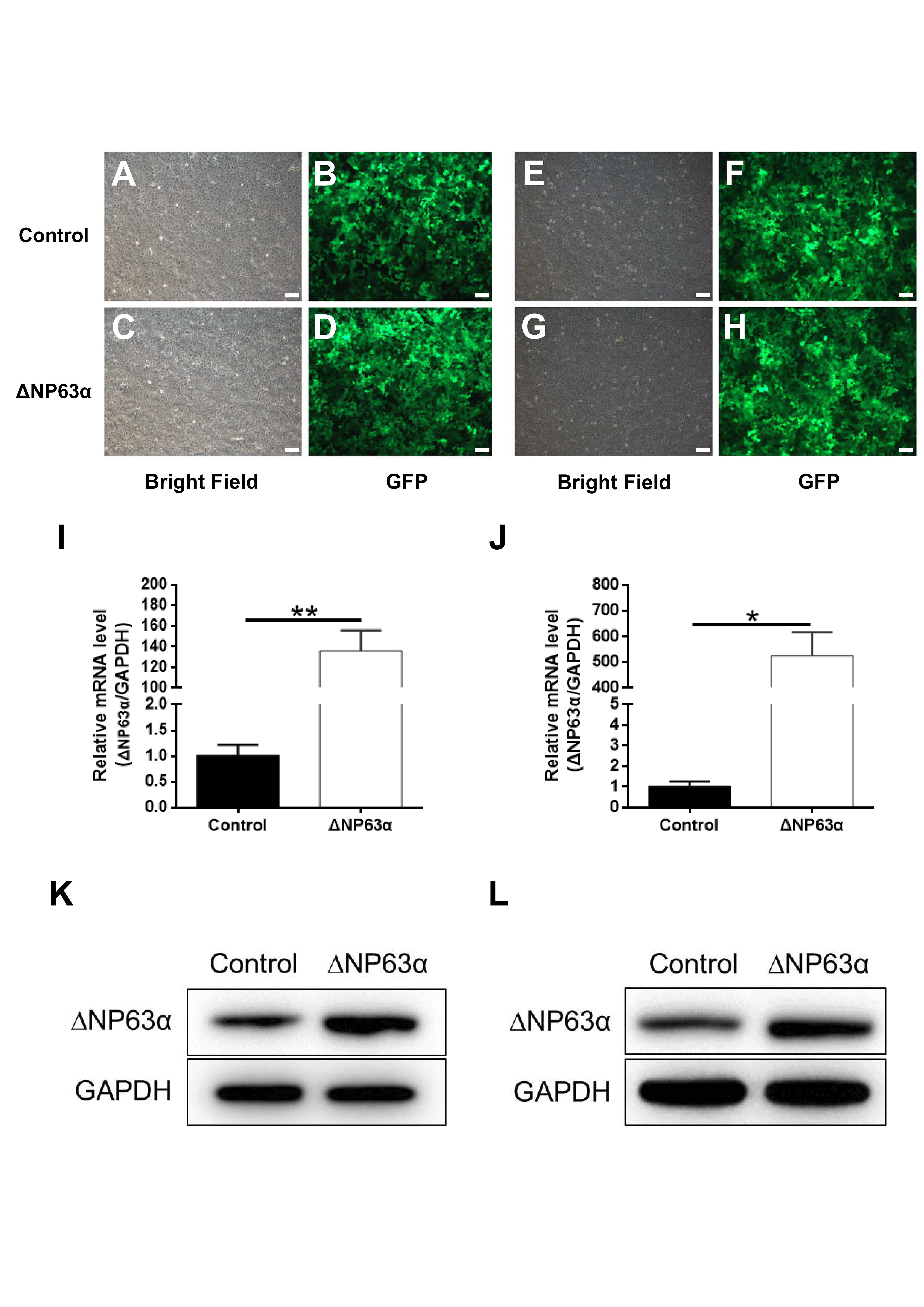

Supplement: Supplementary file 3 — Additional file 3: Figure S1. Establishment of ΔNP63α-overexpressed cells. A–D The fluorescence observation of ΔNP63α-overexpressed HN6 cells. E–H The fluorescence observation of ΔNP63α-overexpressed CAL-27 cells. I The expression levels of ΔNP63α mRNA in ΔNP63α-overexpressed HN6 cells. J The expression levels of ΔNP63α mRNA in ΔNP63α-overexpressed CAL-27 cells. K The expression levels of ΔNP63α protein in ΔNP63α-overexpressed HN6 cells. L The expression levels of ΔNP63α protein in ΔNP63α-overexpressed CAL-27 cells (n = 3). Bars show the mean ± SD. *Represents p < 0.05, **represents p < 0.01. Scale bar = 100 μm. [file 12935_2021_2394_MOESM3_ESM.tif]

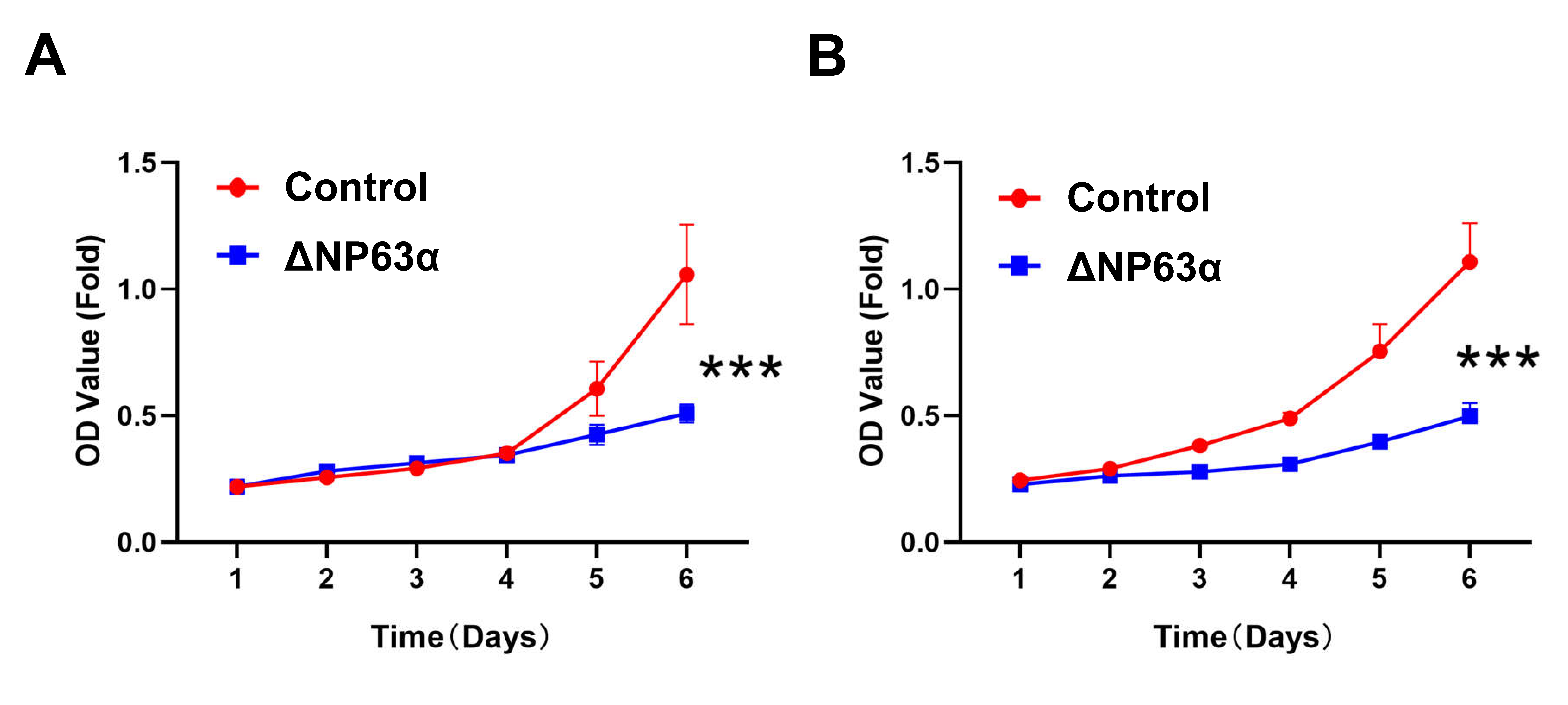

Supplement: Supplementary file 4 — Additional file 4: Figure S2. Cell proliferation analysis of ΔNP63α-overexpressed cells. A MTT assay of HN6 cells. B MTT assay of CAL-27 cells (n = 3). Bars show the mean ± SD. ***Represents p < 0.001. [file 12935_2021_2394_MOESM4_ESM.tiff]

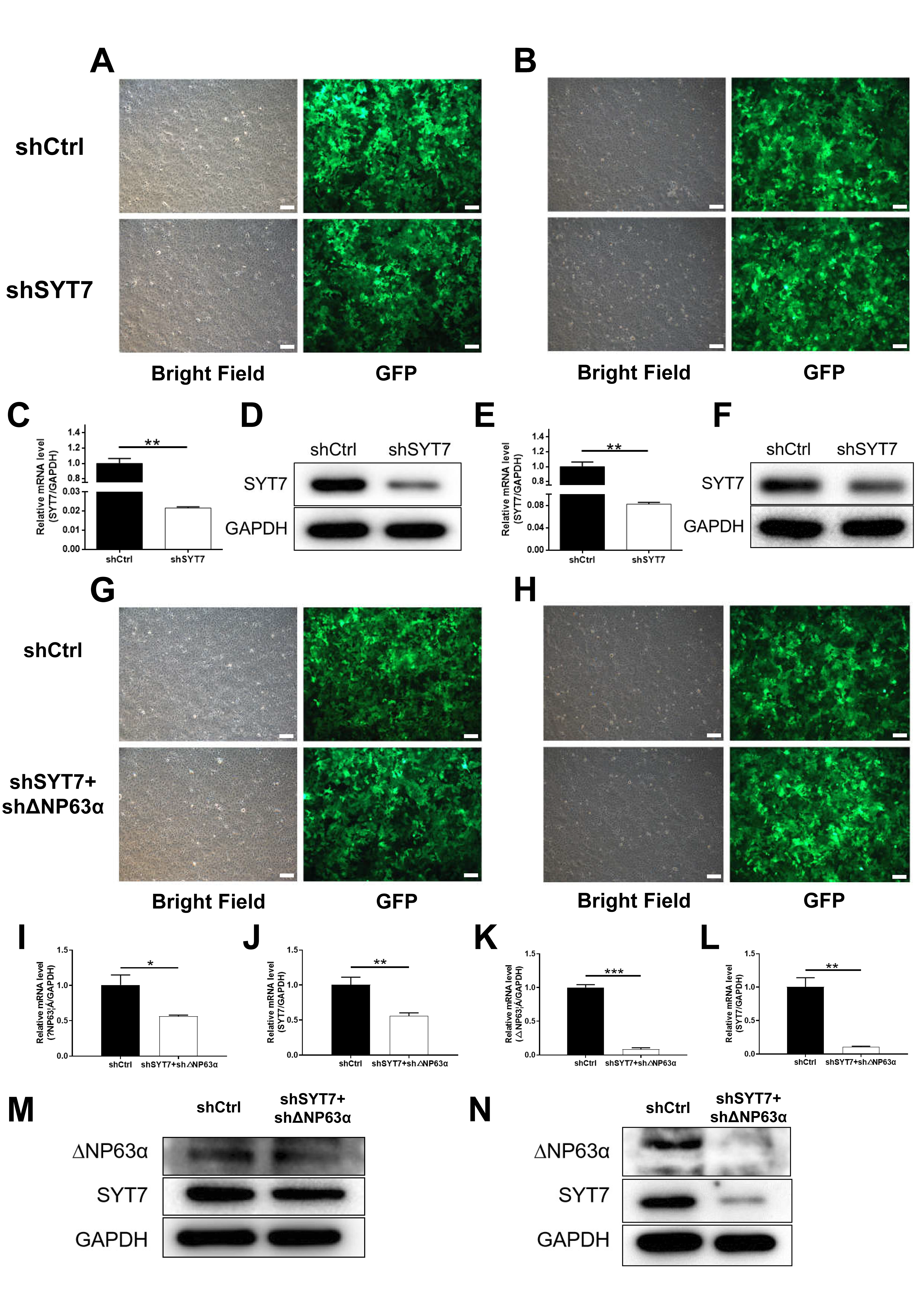

Supplement: Supplementary file 5 — Additional file 5: Figure S3. Establishment of SYT7-knockdown cells and ΔNP63α SYT7 double knockdown cells. A The fluorescence observation of SYT7-knockdown HN6 cells. B The fluorescence observation of SYT7-knockdown CAL-27 cells. C The expression levels of SYT7 mRNA in SYT7-knockdown HN6 cells. D The expression levels of SYT7 protein in SYT7-knockdown HN6 cells. E The expression levels of SYT7 mRNA in SYT7-knockdown CAL-27 cells. F The expression levels of SYT7 protein in SYT7-knockdown CAL-27 cells. G The fluorescence observation of ΔNP63α SYT7 double knockdown HN6 cells. H The fluorescence observation of ΔNP63α SYT7 double knockdown CAL-27 cells. I–J The expression levels of ΔNP63α and SYT7 mRNA in ΔNP63α SYT7 double knockdown HN6 cells. K–L The expression levels of ΔNP63α and SYT7 mRNA in ΔNP63α SYT7 double knockdown CAL-27 cells. M The expression levels of ΔNP63α and SYT7 protein in ΔNP63α SYT7 double knockdown HN6 cells. N The expression levels of ΔNP63α and SYT7 protein in ΔNP63α SYT7 double knockdown CAL-27 cells (n = 3). Bars show the mean ± SD. *Represents p < 0.05, **represents p < 0.01, ***represents p < 0.001. Scale bar = 100 μm. [file 12935_2021_2394_MOESM5_ESM.tif]
